# Fuzzy kernel evidence Random Forest for identifying pseudouridine sites

**DOI:** 10.1093/bib/bbae169

**Published:** 2024-04-15

**Authors:** Mingshuai Chen, Mingai Sun, Xi Su, Prayag Tiwari, Yijie Ding

**Affiliations:** Institute of Fundamental and Frontier Sciences, University of Electronic Science and Technology of China, Chengdu 611731, China; Yangtze Delta Region Institute (Quzhou), University of Electronic Science and Technology of China, Quzhou 324003, China; Beidahuang Industry Group General Hospital, Harbin 150001, China; Foshan Women and Children Hospital, Foshan 528000, China; School of Information Technology, Halmstad University, Sweden; Yangtze Delta Region Institute (Quzhou), University of Electronic Science and Technology of China, Quzhou 324003, China

**Keywords:** RNA sequences, pseudouridine sites, fuzzy feature set, evidence Random Forest, kernel method

## Abstract

Pseudouridine is an RNA modification that is widely distributed in both prokaryotes and eukaryotes, and plays a critical role in numerous biological activities. Despite its importance, the precise identification of pseudouridine sites through experimental approaches poses significant challenges, requiring substantial time and resources.Therefore, there is a growing need for computational techniques that can reliably and quickly identify pseudouridine sites from vast amounts of RNA sequencing data. In this study, we propose fuzzy kernel evidence Random Forest (FKeERF) to identify pseudouridine sites. This method is called PseU-FKeERF, which demonstrates high accuracy in identifying pseudouridine sites from RNA sequencing data. The PseU-FKeERF model selected four RNA feature coding schemes with relatively good performance for feature combination, and then input them into the newly proposed FKeERF method for category prediction. FKeERF not only uses fuzzy logic to expand the original feature space, but also combines kernel methods that are easy to interpret in general for category prediction. Both cross-validation tests and independent tests on benchmark datasets have shown that PseU-FKeERF has better predictive performance than several state-of-the-art methods. This new method not only improves the accuracy of pseudouridine site identification, but also provides a certain reference for disease control and related drug development in the future.

## INTRODUCTION

Pseudouridine ($\Psi $) constitutes a ubiquitous and fundamental RNA modification type, often referred to as “the fifth nucleotide”. This modification has been widely observed in various RNA molecules of both eukaryotic and prokaryotic organisms, including tRNA, mRNA and rRNA [1–3]. Extensive research has revealed that $\Psi $ is essential for several molecular processes, including RNA structural stabilization [4], RNA-protein or RNA-RNA interactions [5], regulating the entry site binding process [6, 7] and participating in RNA metabolis [8, 9]. Moreover, mutations associated with $\Psi $ modification have been discovered to be linked to various diseases, such as lung cancer and atrichia with papular lesions [10–18].

Identifying corresponding $\Psi $ sites is crucial for understanding the mechanism and other functional roles of $\Psi $. However, accurately identifying $\Psi $ sites within RNA sequences poses challenges due to the limitations of current methods. It would be advantageous to develop computational techniques that reliably anticipate $\Psi $ sites from RNA sequence data, such as using machine learning or deep learning methods to build more efficient $\Psi $ site recognition models, which may provide important new perspectives on the function of $\Psi $ based on rapid and accurate recognition capabilities.

Li *et al*. presented PPUS, the first computational technique, in 2015 [19]. It utilized a support vector machine (SVM) [20, 21] and focused on identifying $\Psi $ sites specifically in * S. cerevisiae* and * H. sapiens*. Building upon this, in order to anticipate $\Psi $ sites in *H. sapiens*, *S. cerevisiae* and * M. musculus*, Chen *et al*. developed iRNA-PseU, which integrated nucleotide chemical characteristics with a pseudo nucleotide composition (PseKNC) encoding scheme, trained with SVM [22]. Extensions to the field include the work by He *et al*., who developed PseUI by employing five distinct encoding techniques to extract sequence information from RNA segments. In order to maximize model performance, PseUI, which is also based on SVM, integrated a sequential forward feature selection technique [23]. Tahir *et al*. presented iPseU-CNN, which is a two-layer convolutional neural network model that employed one-hot encoding to forecast $\Psi $ sites [24]. XG-PseU was developed by Liu *et al*. using a forward feature selection technique in conjunction with extreme Gradient Boosting (XGBoost) [25]. An ensemble learning method called EnsemPseU was introduced by Bi *et al*. It integrates techniques from SVM, XGBoost, Naive Bayes, k-nearest neighbor (KNN) and Random Forest (RF) [26]. Using the incremental feature selection process and the light gradient boosting machine (lightGBM), Lv *et al*. created RF-PseU, an RF-based technique [27]. Saad *et al*. presented MU-PseUDeep, a convolutional neural network-based approach that incorporated both sequence and secondary structure features to enhance prediction performance [28]. Li *et al*. subsequently devised Porpoise, a stacked prediction model that selects features from the top four categories and employs them as inputs for predicting $\Psi $ [29]. Additionally, Zhuang *et al*. introduced PseUdeep, a deep learning framework [30]. Wang *et al*. established PsoEL-PseU, a feature fusion predictor, for the identification of $\Psi $ sites [31].While computational methods for predicting $\Psi $ sites have made significant strides, there are still limitations that must be addressed to develop more robust and accurate approaches, as shown in Supplementary Table S1.

**Figure 1:**
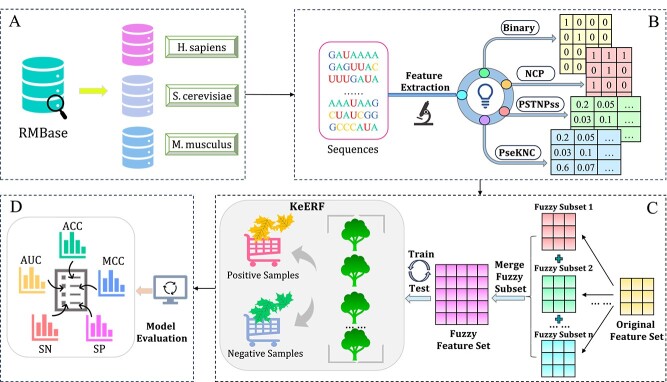
The overall framework of PseU-FKeERF. There are four main steps, including dataset preparation, feature extraction, FKeERF model training and optimization, and performance evaluation.

In this study, we identify $\Psi $ sites via fuzzy kernel evidence Random Forest ( PseU-FKeERF) model in *H. sapiens*, *S. cerevisiae* and *M. musculus*, as shown in Figure 1. First, we test several commonly used RNA sequence coding schemes and selected the best four coding schemes for feature combination. Then, we use fuzzy mean clustering and Gaussian fuzzy membership degree to construct fuzzy feature subset, expand the original feature space, form a new fuzzy feature set, and input it into the KeERF method for category prediction. Finally, we perform cross-validation and independent testing of data sets for each of three species to evaluate performance of model. The results show that the PseU-FKeERF has better predictive performance than other existing models.

## RELATED WORKS

### Fuzzy subset

Fuzzy subset is a set of fuzzy concepts. Given a domain U, then a mapping $\mu _{A}$ from U to the unit interval [0,1] is called a fuzzy subset of U: 


(1) \begin{align*}& \mu_{A}:U\longmapsto \left [ 0,1 \right ]\end{align*}

At present, there are many methods to get fuzzy subset. In TSK-FS [32], when the premise of TSK fuzzy model is determined, the corresponding fuzzy subset is obtained, and its obtaining process is described as follows.

First of all, assume that the training set $x=[x_{1},x_{2},...,x_{m} ]\in R^{n\times m} $ contains m samples and n-dimensional features, where $\ x_{i}={(x_{i1},x_{i2},\ldots ,x_{in})}^{T}\in R^{n\times 1}$. For TSK fuzzy inference system, the most commonly used fuzzy inference rule $R_{k}$ is 


(2) \begin{align*}& \begin{aligned} If\ x_{i1}\ is\ B_{1}^{a}\ \land\ x_{i2}\ is\ B_{2}^{a}\ \land\ \cdots\ \ \land\ x_{in}\ is\ B_{n}^{a},\ \\ Then\ f^{a}\left(x_{i}\right)=p_{0}^{a}+p_{1}^{a}x_{i1}+\cdots+p_{n}^{a}x_{in},\ a=1,\ldots,K \end{aligned}\end{align*}

where $K$ represents the number of fuzzy rules, $B_{j}^{a}$ is the $a$th fuzzy set of the $j$th input feature and $\land $ is a fuzzy association operator. $f^{a}\left (x\right )$ represents the defuzzification function of the local output under the $a$th fuzzy set. The output of TSK fuzzy model can be formulated as 


(3) \begin{align*}& y\left(x_{i}\right)=\sum_{a=1}^{K}\frac{\mu^{a}\left(x_{i}\right)}{\sum_{a^{\prime}=1}^{K}{\mu^{a^{\prime}}\left(x_{i}\right)}}f^{a}\left(x_{i}\right)=\sum_{a=1}^{K}{{\widetilde{\mu}}^{a}\left(x_{i}\right)}f^{a}\left(x_{i}\right)\end{align*}

where $\mu ^{a}\left (x_{i}\right )$ and ${\widetilde{\mu }}^{a}\left (x_{i}\right )$ represent the fuzzy membership and the normalized fuzzy membership associated with the fuzzy set $B^{a}$, respectively, which are calculated by the following formula: 


(4) \begin{align*} & \mu^{a}\left(x_{i}\right)=\prod_{j=1}^{n}{\mu_{B_{i}^{a}}(x_{ij})}\end{align*}

(5) \begin{align*} & {\widetilde{\mu}}^{a}\left(x_{i}\right)=\frac{\mu^{a}\left(x_{i}\right)}{\sum_{a^{\prime}=1}^{K}{\mu^{a^{\prime}}\left(x_{i}\right)}} \end{align*}

where $\mu _{B_{i}^{a}}(x_{ij})$ can be calculated using Gaussian membership function 


(6) \begin{align*}& \mu_{B_{i}^{a}}\left(x_{ij}\right)=\exp{\left(\frac{-\left(x_{ij}-c_{j}^{a}\right)}{2\sigma_{j}^{a}}\right)}\end{align*}

where $c_{j}^{a}$ and $\sigma _{j}^{a}$ are the center and variance of the $a$th fuzzy set in the $j$th dimension, which can be estimated by clustering technique. The FCM algorithm is used to estimate $c_{j}^{a}$ and $\sigma _{j}^{a}$: 


(7) \begin{align*} & c_{j}^{a}=\frac{\sum_{i=1}^{m}{u_{ia}x_{ij}}}{\sum_{i=1}^{m}u_{ia}} \end{align*}

(8) \begin{align*} & \sigma_{j}^{a}=\frac{h\sum_{i=1}^{m}{u_{ia}\left(x_{ij}-c_{j}^{a}\right)^{2}}}{\sum_{i=1}^{m}u_{ia}} \end{align*}

where $u_{ia}$ represents the membership value of sample $x_{i}$ belonging to cluster $a$, and $h$ is the scale parameter, which can be adjusted manually.

After determining the parameters of $c_{j}^{a}$ and $\sigma _{j}^{a}$, let the output of the *a*th fuzzy rule be 


(9) \begin{align*} & {\widetilde{x_{i}}}^{a}={\widetilde{\mu}}^{a}(x_{i})x_{e}, \end{align*}

(10) \begin{align*} & x_{e}=\left(1,\left(x_{i}\right)^{T}\right)^{T}\in R^{(1+n)\times1}. \end{align*}

Thus, the output of $K$ fuzzy rules can be represented as follows: 


(11) \begin{align*} & x_{gi}=\left(\left({\widetilde{x_{i}}}^{1}\right)^{T},\left({\widetilde{x_{i}}}^{2}\right)^{T},\ldots,\left({\widetilde{x_{i}}}^{K}\right)^{T}\right)^{T}\in R^{\left[\left(1+n\right)\times K\right]\times1}, \end{align*}

(12) \begin{align*} & x_{g}=\left(x_{g1},x_{g2},\ldots,x_{gi},\ldots,x_{gm}\right)^{T}\in R^{m\times\left[\left(1+n\right)\times K\right]} \end{align*}

where $x_{g}$ is the final fuzzy subset, which enlarges the original feature space.

### Dempster–Shafer evidence theory

Famous academics Dempster and Shafer created the D-S evidence theory [33, 34]. Suppose there is a problem that needs to be decided, and the complete set of all possible results is denoted by $\Theta $, and there is a mutually exclusive relationship between all elements of $\Theta $. The set $\Theta $ is called the recognition frame and can be expressed as: 


(13) \begin{align*}& \Theta=\left\{\theta_{1},\theta_{2},\ldots,\theta_{N}\right\}\end{align*}

where $\theta _{j}$ is an element of the recognition frame $\Theta $. The set $2^{\Theta }$ of all subsets under the identification frame $\Theta $ is expressed as 


(14) \begin{align*}& 2^{\Theta}=\left\{\Phi,\ \left\{\theta_{1}\right\},\left\{\theta_{2}\right\},\ldots,\left\{\theta_{1}\cup\theta_{2}\right\},\ldots,\Theta\right\}\end{align*}

After determining the identification framework, evidence theory uses the basic trust distribution function to systematically summarize the final distribution results of all propositions. The basic trust assignment function m on the identification frame $\Theta $ is a mapping of $2^{\Theta }\longmapsto [0,\ 1]$, which satisfies the following conditions: 


(15) \begin{align*}& \left\{\begin{matrix}m\left(\phi\right)=0 \\ \sum_{D\subseteq\Theta}{m(D)=1} \end{matrix}\right.\end{align*}

where $m(D)$ represents the degree of support of evidence for proposition $D$, and its value is the proposition’s fundamental trust assignment value. The empty set has a base trust value of zero, and the total trust value of all other subsets is equal to one. $D$ is referred to as a focal element if the value of $m(D)$ exceeds 0.

Dempster proposed a method of evidence synthesis, that is, the basic trust allocation function of two or more evidences is calculated in the way of orthogonal sum, which is called Dempster synthesis rule. Suppose that under the same recognition framework $\Theta $, there are n groups of evidence, $m_{1},m_{2},\ldots ,m_{n}$ is the basic trust assignment function corresponding to each evidence, and the focal elements are $D_{1},D_{2},\ldots ,D_{n}$, Dempster’s composition rule is as follows: 


(16) \begin{align*} & m(D)=\left\{\begin{matrix}\frac{\sum_{D_{1}\cap\ldots\cap D_{n}=D}\prod_{1\le i\le n}{m_{i}\left(D_{i}\right)}}{1-K},\ D\neq\Phi \\ 0,D=\Phi \end{matrix}\right. \end{align*}

(17) \begin{align*} & K=\sum_{D_{1}\cap\ldots\cap D_{n}=\Phi}\prod_{1\le i\le n}{m_{i}\left(D_{i}\right)}. \end{align*}

The equation is unstable when used in the prediction of a large number of estimators. Therefore, for predictions from a large number of different estimators, a simple average of the mass function is preferred, which is defined as follows: 


(18) \begin{align*}& m\left(D\right)=\frac{1}{N}\sum_{i=1}^{N}{m_{i}\left(D\right),D\in}2^{\Theta}.\end{align*}

### The Jousselme distance approach

Evidence distance is used to represent the similarity between evidence. Jousselme *et al*. proposed Jousselme distance [35] by using the geometric interpretation of evidence theory.

Assuming that the recognition frame $\Theta $ contains several elements, a high-dimensional space can be obtained by taking the elements in $2^{\Theta }$ as coordinates. Each evidence can be represented as a vector in the higher-dimensional space. If $m_{f}$ and $m_{k}$ are two independent evidences in the recognition framework, the evidence is represented as a vector in the space, denoted as $\vec{m_{f}}$ and $\vec{m_{k}}$. The Jousselme distance between $m_{f}$ and $m_{k}$ is defined as: 


(19) \begin{align*}& d_{J}\left(m_{f},m_{k}\right)=\sqrt{\frac{1}{2}\left(m_{f}-m_{k}\right)^{T}D\left(m_{f}-m_{k}\right)},\end{align*}

where $D$ is a $2^{N}\times 2^{N}$ symmetric matrix, and the elements in the matrix are 


(20) \begin{align*}& d_{ij}=\frac{\left|A_{i}\cap B_{j}\right|}{\left|A_{i}\cup B_{j}\right|},\ {\ \ \ \ A}_{i},B_{j}\in\Theta.\end{align*}

The ratio of intersection and union of focal elements $A_{i}$ and $B_{j}$ is used to express their similarity, which is called Jaccard coefficient.

### The inclusion degree

Martin defines two inclusions [36] to measure how two mass functions are contained, one for strict inclusion and the other for light inclusion. Strict inclusion requires that the mass function $m_{a}$ is contained in $m_{b}$ if all focal elements of $m_{a}$ are contained in every focal element of $m_{b}$. A strict degree of inclusion of $m_{a}$ in $m_{b}$ is given by 


(21) \begin{align*} & \delta_{S}\left(m_{a},m_{b}\right)=\frac{1}{\left|\mathcal{F}_{a}\right|\left|\mathcal{F}_{b}\right|}\sum_{X_{a}\in\mathcal{F}_{a}}\sum_{Y_{b}\in\mathcal{F}_{b}}{Inc\left(X_{a},Y_{b}\right)} \end{align*}

(22) \begin{align*} & Inc\left(X_{a},Y_{b}\right)=1\ if\ X_{a}\subseteq Y_{b}\ and\ 0\ otherwise \end{align*}

where $\mathcal{F}_{a}$ and $\mathcal{F}_{b}$, respectively, are the set of focal elements of $m_{a}$ and $m_{b}$, and $\left |\mathcal{F}_{a}\right |,\left |\mathcal{F}_{b}\right |$ are the number of focal elements of $m_{a}$ and $m_{b}$.

Light inclusion says that the mass function $m_{a}$ is contained in $m_{b}$ if all focal elements of $m_{a}$ are contained in at least one focal element of $m_{b}$. It is defined as follows: 


(23) \begin{align*}& \delta_{L}\left(m_{a},m_{b}\right)=\frac{1}{\left|\mathcal{F}_{a}\right|}\sum_{X_{a}\in\mathcal{F}_{a}}\max_{Y_{b}\in\mathcal{F}_{b}}{Inc\left(X_{a},Y_{b}\right)}.\end{align*}

On the basis of strict inclusion, Hoarau defines a new type of inclusion called the fair inclusion. The fair inclusion is defined as the mass function $m_{a}$ being fairly included in $m_{b}$ if all focal elements of $m_{a}$ on $2^{\Theta } \setminus \Theta $ are, in turn, included in each focal element of $m_{b}$ on $2^{\Theta } \setminus \Theta $. It is given as follows: 


(24) \begin{align*}& \delta_{F}\left(m_{a},m_{b}\right)=\frac{1}{\left|\mathcal{L}_{a}\right|\left|\mathcal{L}_{b}\right|}\sum_{X_{a}\in\mathcal{L}_{a}}\sum_{Y_{b}\in\mathcal{L}_{b}}{Inc\left(X_{a},Y_{b}\right)}\end{align*}

where $\mathcal{L}_{a}$ and $\mathcal{L}_{b}$ represent the set of focal elements of $\mathcal{L}_{a}$ and $\mathcal{L}_{b}$ on $2^{\Theta } \setminus \Theta $, respectively. The fair inclusion is a slightly less strict degree of inclusion than strict inclusion, because ignorance is contained only in itself.

On the basis of the integration of the above techniques, we improved the method to build a better classification performance of $\Psi $ sites recognition model.

## METHODS

### Feature extraction

In this study, we comprehensively tested multiple RNA sequence coding schemes. According to their predictive performance, the best four coding schemes were selected to determine the best feature combination, including position-specific trinucleotide propensity based on single-strand (PSTNPss). nucleotide chemical property (NCP), pseudo k-tuple composition (PseKNC) and binary feature [37–43].

#### Binary feature

Four-dimensional binary vectors are utilized in the binary feature to represent nucleotides. For instance, the RNA bases A, C, G and U are represented as $(1\ 0\ 0\ 0)$, $(0\ 1\ 0\ 0)$, $(0\ 0\ 1\ 0)$ and $(0\ 0\ 0\ 1)$, respectively.

#### Nucleotide chemical property

Chemical structures and characteristics vary throughout nucleotides. Table 1 illustrates how four nucleotides may be grouped into three distinct groups based on their chemical characteristics.

**Table 1:** Chemical properties of four nucleotide types

Chemical property	Class	Nucleotides
Ring structure	Purine	A, G
	Pyrimidine	C, U
Functional group	Amino	A, C
	Keto	G, U
Hydrogen bond	Strong	C, G
	Weak	A, U

We may utilize three-dimensional coordinates to encode A, C, G and U based on their various chemical characteristics. They are encoded as $(1\ 1\ 1)$, $(0\ 1\ 0)$, $(1\ 0\ 0)$ and $(0\ 0\ 1)$, respectively.

#### Position-specific trinucleotide propensity based on single strand

RNA sequences are encoded by the PSTNPss utilizing statistical rules. 64 trinucleotides were typically present (e.g., ‘AAA’, ‘AAC’, ‘AAG’, $\ldots $, ‘UUU’). Consequently, a $64\times (L-2)$ matrix defines the position specificity of a trinucleotide for a particular RNA sequence of length $L-bp$: 


(25) \begin{align*}& Z=\left[\begin{matrix}z_{1,1}&\cdots&z_{1,L-2}\\\vdots&\ddots&\vdots\\z_{64,1}&\cdots&z_{64,L-2}\\\end{matrix}\right]_{64\times\left(L-2\right)}\end{align*}

where 


(26) \begin{align*}& \begin{aligned} Z_{m,n}=F^{+}\left({3mer}_{s}\middle| t\right)-F^{-}\left({3mer}_{s}\middle| t\right), \\ \ s=1,2,\ldots,64,\ t=1,2,\ldots,L-2. \end{aligned}\end{align*}

$F^{+}\left ({3mer}_{s}\middle |\ t\right )$ and $F^{-}\left ({3mer}_{s}\middle |\ t\right )$, respectively, represent the frequencies of the $s$th trinucleotide $({3mer}_{m})$ at the $t$th position appearing in the positive $(D^{+})$ and negative $(D^{-})$ datasets, where ${3mer}_{1}=\mathrm{^{\prime}AAA^{\prime}},3mer_{2}=\mathrm{^{\prime}AAC^{\prime}},\ldots ,3mer_{64}=\mathrm{^{\prime}UUU^{\prime}} $. Therefore, a given RNA sequence of $L-bp$ length can be denoted as follows: 


(27) \begin{align*}& D=\left[\vartheta_{1},\vartheta_{2},\ldots,\vartheta_{L-2}\right]^{T},\end{align*}

where $\vartheta _{t}$ is expressed as: 


(28) \begin{align*}& v_{t} =\left\{\begin{matrix}d_{1,t},where\ N_{t}N_{t+1}N_{t+2}=AAA \\... \\d_{64,t},where\ N_{t}N_{t+1}N_{t+2}=UUU \end{matrix}\right.\end{align*}

#### Pseudo k-tuple composition

One kind of pseudo nucleic acid composition feature that takes into account both local and long-range sequence information is the PseKNC feature. PseKNC is defined as follows: 


(29) \begin{align*}& A=\left[a_{1},a_{2},\ldots,a_{4^{k}},a_{4^{k}+1},\ldots,a_{4^{k}+\lambda}\right]^{T}\end{align*}

where 


(30) \begin{align*}& \alpha_{k} =\left\{\begin{matrix}\frac{f_{\alpha}}{\sum_{m=1}^{4^{k}}{f_{m}+\omega\sum_{n=1}^{\lambda}\theta_{n}}},\left(1\le\alpha\le4\right) \\ \frac{\omega\theta_{\alpha-4^{k}}}{\sum_{m=1}^{4^{k}}{f_{m}+\omega\sum_{n=1}^{\lambda}\theta_{n}}},\left(4^{k}\le\alpha\le4^{k}+\lambda\right) \end{matrix}\right.\end{align*}

where $f_{\alpha }\left (\alpha =1,2,\ldots ,4^{k}\right )$ represents the frequency of oligonucleotides, $\omega $ represents the factor and $\theta _{n}$ is defined as follows: 


(31) \begin{align*}& \begin{aligned} \theta_{n}=\frac{1}{L-n-1}\sum_{m=1}^{L-n-1}\phi\left(R_{m}R_{m+1},R_{m+n}R_{m+n+1}\right), \\ (n=1,2,\ldots,\lambda;\lambda<L). \end{aligned}\end{align*}

$\phi \left (R_{m}R_{m+1},R_{m+n}R_{m+n+1}\right )$ is the correlation function, which is defined as: 


(32) \begin{align*}& \begin{aligned} \phi\left(R_{m}R_{m+1},R_{m+n}R_{m+n+1}\right) =\frac{1}{\sigma}\sum_{\xi=1}^{\sigma}[P_{\xi}\left(R_{m}R_{m+1}\right)- \\ P_{\xi}\left(R_{m+n}R_{m+n+1}\right)]^{2} \end{aligned}\end{align*}

where $\sigma $ represents the number of physicochemical indices and $P_{\xi }\left (R_{m}R_{m+1}\right )$ is the value of the $\xi $th ($\xi =1,2,\ldots ,\sigma $) physicochemical index of dinucleotide $R_{m}R_{m+1}$ at the position *m*.

### Evidence RF model based on fuzzy logic and kernel method

**Figure 2:**
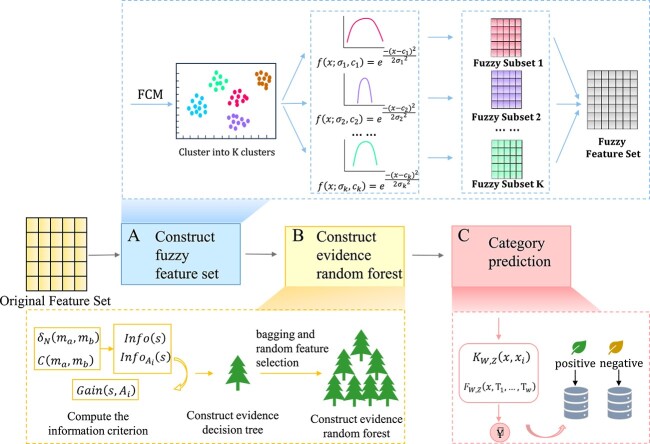
The flowchart of FKeERF method. FKeERF method has three parts, including constructing fuzzy feature set, constructing evidence decision tree and category prediction. (**A**) By clustering the original feature set through fuzzy means, several clusters and the mean and variance of each cluster are obtained. Then, multiple fuzzy feature subsets are obtained by using Gaussian membership function, and fuzzy feature sets are obtained by fusing multiple fuzzy feature subsets. (**B**) Use fuzzy feature set to construct evidence random forest. (**C**) Input the training set and test set samples into the evidence random forest, respectively, count the number of the two falling on the same node, use the kernel function and prediction function to obtain the prediction result, and combine the symbol function to obtain the final prediction label.

We construct an evidence RF model based on fuzzy logic and kernel methods, as shown in Figure 2. The following is the specific construction process of the model.

First of all, since the dimension of the original feature set obtained after feature extraction is small, in order to further improve the recognition performance of the subsequent model, the implementation idea of fuzzy subset is obtained by referring to TSK-FS, the original feature set is processed by fuzzy logic to obtain multiple fuzzy subsets, and a new fuzzy feature set is obtained by merging all fuzzy subsets. 


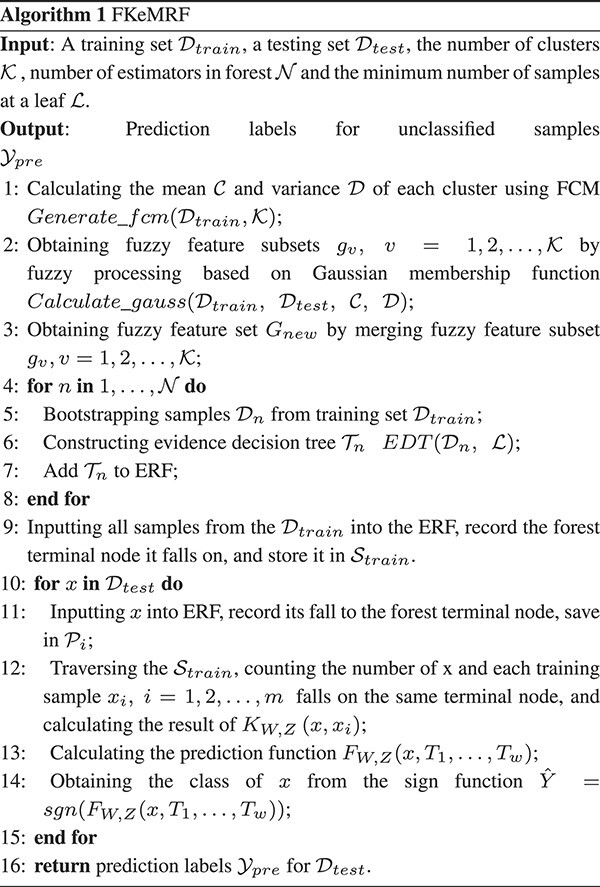


Then, the new fuzzy feature set is input into the evidence RF classifier based on kernel method (KeERF) for training. The construction of KeERF is divided into the following two steps:

(i) Construct evidence decision trees by using conflict concept in belief function theory.

(ii) Using bagging and random feature selection on the evidence decision tree to obtain evidence RF.

Finally, the kernel method is used to predict the category labels of unclassified samples. The FKeERF method is summarized and introduced in detail in Algorithm 1. The following formula symbols are described in Table 2.

**Table 2:** Description of symbols in FKeERF model formulas

Notation	Description
$\delta ^{a\subseteq b}\left (m_{a},m_{b}\right )$	The fair degree of inclusion
$\delta _{N}\left (m_{a},m_{b}\right )$	The degree of inclusion of $m_{a}$ and $m_{b}$
$C\left (m_{a},m_{b}\right )$	The conflict measure of $m_{a}$ and $m_{b}$
${Info}\left (s\right )$	The information of node s
${Info}_{A_{i}}\left (s\right )$	The weighted sum of the information of the child nodes split on the attribute $A_{i}$
$Gain(s,A_{i})$	The split information gain on attribute $A_{i}$
$m\left (D\right )$	The mass function value of class $\omega $ in proposition $D$
$Bet\ P\left (\omega \right )$	The pignistic probability of the mass function
$K_{W,Z}\left (x,x_{i}\right )$	The posterior probability
$F_{W,Z}(x,T_{1},\ldots ,T_{w})$	The prediction function
$\hat{Y}$	The predicted label value

#### Evidential decision trees

Unlike traditional decision trees, the evidence decision tree proposed by Hoarau *et al*. improves the node splitting criteria [44]. They propose to use a conflict measure based on inclusion and Jousselme distance as a split criteria. First, based on fair inclusion, the inclusion of $m_{a}$ and $m_{b}$ is defined as: 


(33) \begin{align*}& \delta_{N}\left(m_{a},m_{b}\right)=\max{\left(\delta^{a\subseteq b}\left(m_{a},m_{b}\right),\delta^{b\subseteq a}\left(m_{b},m_{a}\right)\right)}.\end{align*}

This inclusion gives the maximum proportion of focus elements in one mass function in the other. The conflict measure of $m_{a}$ and $m_{b}$ is expressed as: 


(34) \begin{align*}& C\left(m_{a},m_{b}\right)=\left(1-\delta_{N}\left(m_{a},m_{b}\right)\right)d_{J}\left(m_{a},m_{b}\right),\end{align*}

which is used as the node splitting criterion in the evidence decision tree. According to the collision measurement, the information $Info(s)$ of node $s$ is defined as follows: 


(35) \begin{align*}& Info\left(s\right)=\frac{\sum_{x_{a}\in s}\sum_{x_{b}\in s} C\left(m_{a},m_{b}\right)}{\left|s\right|^{2}-\left|s\right|}.\end{align*}

Let node $s$ be a set of observations. Let $Attr={A_{1},A_{2},...\ A_{m}}$ be its attribute set, then the split information gain $Gain(s,A_{i})$ on attribute $A_{i}$ is 


(36) \begin{align*}& Gain\left(s,A_{i}\right)=Info\left(s\right)-{Info}_{A_{i}}\left(s\right),\end{align*}

where $Info\left (s\right )$ is the information of the node $s$ and ${Info}_{A_{i}}\left (s\right )$ is the weighted sum of the information of the child nodes split on the attribute $A_{i}$, which is expressed as: 


(37) \begin{align*}& {Info}_{A_{i}}\left(s\right)=\sum_{v\in A_{i}}\frac{\left|s_{v}\right|}{\left|s\right|}Info\left(s_{v}\right).\end{align*}

$s_{v}$ is the subset of s for which attribute $A_{i}$ has the value $v$. Select the attribute that maximize the gain function for splitting a node. Until the stop criteria are satisfied, splits are carried out recursively for every child node, beginning at the root of each node.

Once the tree is constructed, a new observation will pass through the tree from the root based on the value of its attribute. The observation will be given a mass function equal to the node’s average mass function once a leaf is reached.

In evidence theory, decisions are made based on the resulting pignistic probabilities by converting mass functions into pignistic probabilities. Therefore, the class that maximizes the pignistic probability $Bet\ P(\omega )$ of this mass function is the prediction class: 


(38) \begin{align*}& Bet\ P\left(\omega\right)=\sum_{D\in2^{\Theta},\ \omega\in D}\frac{m\left(D\right)}{\left|D\right|}\end{align*}

where $\omega $ represents the class, $m(D)$ is the mass function value of class $\omega $ in proposition $D$, and $|D|$ represents the number of mass functions of proposition $D$.

#### Evidence RF based on kernel method

Based on evidence decision tree, bagging and random feature selection are used to construct evidential RFs. After completing the construction of the evidence RF, for unclassified samples, instead of directly inputting them into the RF to obtain the corresponding label as in traditional prediction methods, the prediction will be carried out using the kernel method [45, 46].

Firstly, the posterior probability $K_{W,Z}\left (x,x_{i}\right )$ that the unclassified sample $x$ and all training samples $X_{train}=\{x_{1},x_{2},\ldots ,x_{i},\ldots ,x_{Q}\}$ fall on the same leaf node in the evidence RF is calculated: 


(39) \begin{align*}& K_{W,Z}\left(x,x_{i}\right)=\frac{1}{W}\sum_{w=1}^{W} 1_{x_{i}\in I_{z(x,T_{w})}}\end{align*}

where $W$ is the number of evidence decision trees in the evidence RF, $T_{w}$ represents the split mode of evidence decision tree in the $w^{th}$ iteration, and $I_{z(x,T_{w})}$ is defined as a node containing $x$ in the evidence RF, which is determined by $T_{w}$ and the training set $X_{train}$. When $x_{i}$ and $x$ are connected to the same leaf node in the evidence decision tree model, the value of $1_{x_{i}\in I_{z(x,T_{w})}}$ is equal to 1.

Then use the prediction function $F_{W,Z}(x,T_{1},\dots ,T_{w})$ to obtain an approximate prediction value 


(40) \begin{align*}& F_{W,Z}(x,T_{1},\ldots,T_{w})=\frac{\sum_{i=1}^{Z}{y_{i}K_{W,Z}\left(x,x_{i}\right)}}{\sum_{l=1}^{Z}{K_{W,Z}\left(x,x_{l}\right)}}.\end{align*}

Finally, according to the approximate predicted value, the label value is predicted by using the symbol function 


(41) \begin{align*}& \begin{aligned} \hat{Y}=sgn(F_{W,Z}(x,T_{1},\ldots,T_{w}))= \\ \left\{\begin{matrix}1,F_{W,Z}(x,T_{1},\ldots,T_{w})>0 \\ -1,F_{W,Z}(x,T_{1},\ldots,T_{w})<0 \end{matrix}\right.. \end{aligned}\end{align*}

## RESULTS

The following experiments were run on a server equipped with an Intel Xeon Platinum 8168 CPU and 1.0 TB of memory. We trained the model using the Python programming language and experimented with the Ubuntu 16.04.1 LTS operating system.

### Datasets

The benchmark dataset collected from RMBase [47] by Chen *et al*. [22] is used for comparison, as shown in Table 3. These datasets consist of three training datasets, $H\_990$, $S\_628$ and $M\_944$, and two separate test datasets, $H\_200$ and $S\_200$. The sample sizes of the three training datasets are 990 628 and 944, with half of each dataset containing $\Psi $ sites and the other half not. Both independent testing datasets contain 200 samples, half of which contain $\Psi $ sites. In both the *H. sapiens* dataset ($H\_990$ and $H\_200$) and the *M. musculus* dataset ($M\_944$), all samples exhibit RNA sequences consisting of 21 nucleotides with uridine positioned at the center. Conversely, within the *S. cerevisiae* datasets ($S\_628$ and $S\_200$), the RNA sequences encompass 31 nucleotides with uridine occupying the central position. All datasets used in the experiment are existing datasets. In order to ensure the validity of the results comparison, we used the same dataset as other comparison models.

**Table 3:** The information of benchmark datasets

Species	Datasets	Length	Positive samples	Negative samples
*H. sapiens*	H_990	21 bp	495	495
	H_200	21 bp	100	100
*S. cerevisiae*	S_628	31 bp	314	314
	S_200	31 bp	100	100
*M. musculus*	M_944	21 bp	472	472

### Evaluation metrics

This study presents a new method for identifying $\Psi $ sites. We evaluated the performance of our method using five metrics widely used in previous studies: specificity (SP), sensitivity (SN), accuracy (ACC), Matthew correlation coefficient (MCC), F1 score, area under the precision-recall curve (AUPR) and area under the receiver operating characteristic curve (AUC) [48–55]. The AUC (Area Under Curve) is defined as the area under the ROC curve and enclosed by a coordinate axis, and its value ranges between 0.5 and 1. The closer the AUC is to 1.0, the higher the authenticity of the detection method. The formula for these metrics is as follows: 


(42) \begin{align*} & \ SP=\frac{TN}{TN+FP} \end{align*}

(43) \begin{align*} & SN=\frac{TP}{FN+TP} \end{align*}

(44) \begin{align*} & ACC=\frac{TP+TN}{TP+TN+FP+FN} \end{align*}

(45) \begin{align*} & MCC= \frac{TN\times T P-FN\times F P}{\sqrt{\left(TP+FP\right)\left(TP+FN\right)\left(TN+FN\right)\left(TN+FP\right)}} \end{align*}

(46) \begin{align*} & Precision = \frac{TP}{TP+FP} \end{align*}

(47) \begin{align*} & F1 = \frac{2\times Precision \times SN}{Precision + SN} \end{align*}

where TP represents true positive, FP represents false positive, TN represents true negative, FN represents false negative. We use these metrics to evaluate the performance of our methods and guide our experiments. Independent testing experiment refers to applying a model trained on a training dataset to an independent testing dataset to evaluate the generalization ability of the model.

### Performance evaluation of different feature extraction schemes

**Figure 3:**
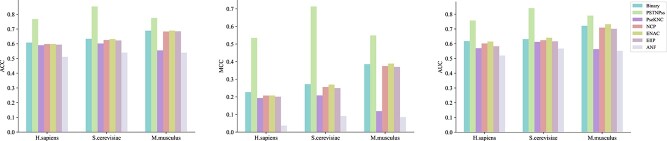
Comparison of different feature extraction schemes.

**Figure 4:**
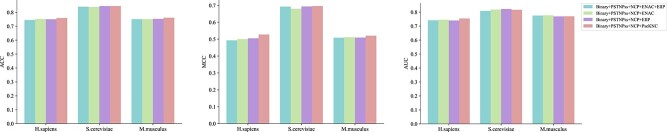
Comparison of different feature extraction scheme combinations.

In this section, we thoroughly investigate and evaluate the performance of seven different feature encodings including Binary, NCP, ENAC, PseKNC, EIIP, PSTNPss and ANF by conducting 10-fold cross-validation tests and identify the best combination of features. First, we extracted features from the training sets of the three species according to seven coding schemes respectively, and then selected RF as a classifier to conduct 10-fold cross-validation tests to obtain the performance results of each coding scheme, as shown in Figure 3. Through comprehensive analysis of the test results of the three training sets, it can be seen that PSTNPss has the best performance, Binary, NCP, ENAC, PseKNC and EIIP follow, and ANF has the worst performance. Therefore, we choose the remaining six encoding schemes besides ANF for feature combination. According to the performance of each coding scheme in the previous stage, we designed four feature combination modes and input them into the RF classifier for testing. Finally, the performance results of the four feature combination schemes were obtained, as shown in Figure 4. Through comprehensive analysis, it can be seen that the combination of Binary, PSTNPss, NCP and PseKNC has the best performance.

After testing single features and feature combinations, we finally selected Binary, PSTNPss, NCP and PseKNC feature combinations for subsequent experiments

### Parameter optimization and analysis

Hyperparameters affect the recognition performance of the model, and other advanced methods optimize the hyperparameters to some extent, as shown in the Supplementary Table S2. We also discuss the role of hyperparameters in PseU-FKeERF: (i) The number of clusters in fuzzy mean clustering; (ii) construct the number of decision trees of the forest; (iii) minimum number of leaf node samples. Parameter analysis experiments were carried out on the training datasets of three species.

**Figure 5:**
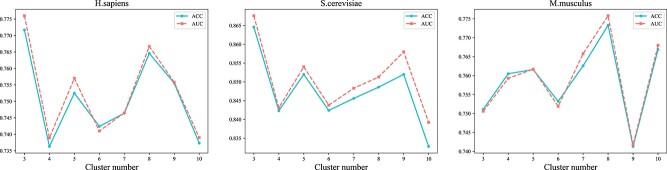
Performance comparison under different cluster number.

In the process of constructing fuzzy feature set, it is necessary to carry out fuzzy mean clustering first. The number of clusters determines the number of fuzzy subsets, which is an important hyperparameter that affects the performance of site recognition model. Therefore, we preliminarily set the number of clusters from 3 to 10, with an interval of 1, for testing. Figure 5 shows the performance results of the model under different cluster numbers when the number of decision trees is 100 and the minimum leaf node sample number is 5. As shown in the figure, when the cluster number is set to 3, our model achieves high performance on the *H. sapiens* and *S. cerevisiae* training sets. For the *M. musculus* training set, the performance is better when the number of clusters is 8.

**Figure 6:**
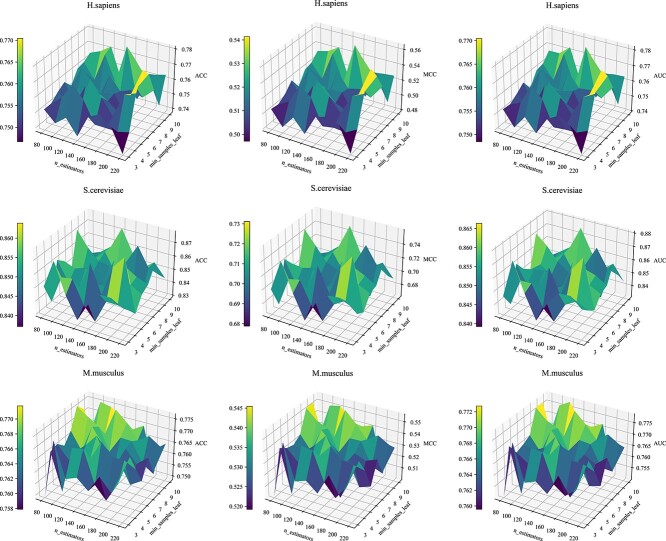
Parameter setting analysis of decision tree number and minimum leaf node sample number in FKeERF model on three species datasets.

We then used grid search to estimate the effects of two additional hyperparameters: the number of decision trees with search ranges from 80 to 220 at intervals of 20 and the number of samples of the smallest leaf nodes with search ranges from 3 to 10 at intervals of 1. Figure 6 shows the performance results when the two hyperparameters are set to different values when the cluster number is 3. As shown in the figure, when the number of decision trees is 140 and the minimum sample number of leaf nodes is 10, the model performs best on the *H. sapiens* training set. For *S. cerevisiae*, the accuracy of the model is the highest when the number of decision trees is 120 and the minimum leaf node sample is 9. The *M. musculus* training set has the best performance when the number of decision trees is 200 and the minimum sample number of leaf nodes is 6.

In general, based on the above analysis of experimental results, we determined the optimal parameter setting of the PseU-FKeERF model on each species dataset, including the number of clusters, the number of decision trees and the number of minimum leaf node samples, which are crucial for achieving the best model performance.

### Ablation study

To verify the contribution and validity of each module in the FKeERF method, we conducted ablation studies on training sets and independent testing sets for three species. We perform the ablation experiment by removing the kernel method to predict the class module and the fuzzy subset module. Specifically, the first method, ERF, does not add the kernel method prediction category module and the fuzzy subset module, that is, the original evidence RF method. The second method, KeERF, is to add only the kernel method prediction category module and use the kernel method to predict the category label of each unclassified sample. The third method FKeERF is to add fuzzy subset module on the basis of KeERF. By combining fuzzy logic rules, multiple fuzzy subsets are fused to form fuzzy feature set and expand the original feature space.

The final test results are shown in Tables 4 and 5, respectively. Compared with the other two methods, FKeERF has the best performance. The comparison results of the first two methods show that removing the category prediction module of the kernel method will reduce the prediction performance of the model, which indicates that introducing the kernel method to improve the category label prediction is helpful to improve the prediction performance of the model. For the ablation of fuzzy subset modules, the results show that our method FKeERF expands the original feature space by combining fuzzy logic rules, and further improves the prediction performance.

**Table 4:** Results of ablation study on three species training datasets

Species	Classifier	10-fold cross-validation				
		ACC	MCC	SN	SP	AUC
*H. sapiens*	ERF	0.7383	0.4787	0.7303	0.7501	0.7402
	KeERF	0.7464	0.4965	0.7466	0.7494	0.748
	FKeERF	0.7808	0.5644	0.7762	0.7866	0.7814
*S. cerevisiae*	ERF	0.8504	0.7078	0.8314	0.8731	0.8522
	KeERF	0.8583	0.7266	0.858	0.875	0.8665
	FKeERF	0.8773	0.7589	0.8608	0.8998	0.8803
*M. musculus*	ERF	0.7692	0.5405	0.7764	0.7651	0.7707
	KeERF	0.7722	0.5486	0.7842	0.7649	0.7745
	FKeERF	0.7787	0.5571	0.7785	0.7782	0.7784

**Table 5:** Results of ablation study on two species independent testing datasets

Species	Classifier	Independent testing				
		ACC	MCC	SN	SP	AUC
*H. sapiens*	ERF	0.89	0.7827	0.8629	0.9168	0.8899
	KeERF	0.8949	0.7855	0.8919	0.8948	0.8934
	FKeERF	0.9199	0.8504	0.9011	0.9497	0.9254
*S. cerevisiae*	ERF	0.92	0.8396	0.9231	0.9199	0.9215
	KeERF	0.9349	0.8779	0.9123	0.9602	0.9363
	FKeERF	0.945	0.8898	0.9329	0.9608	0.9468

In general, the ablation experiments show that the kernel method prediction category module and fuzzy subset module in our method are effective for improving the prediction performance.

### Comparison with traditional machine learning methods

We compare our model with RF, SVM, KNN, XGBoost and other methods to further confirm the performance of our approach, FKeERF. We tested the predictive performance of all classifiers in the three species training sets and independent testing sets, and the results are shown in Tables 6 and 7.

**Table 6:** Performance of different classifiers on three species training datasets

Species	Classifier	10-fold cross-validation				
		ACC	MCC	SN	SP	AUC
*H. sapiens*	KNN	0.606	0.2232	0.6043	0.6208	0.6125
	SVM	0.6	0.2053	0.6035	0.6019	0.6027
	XGBoost	0.7373	0.4765	0.738	0.7406	0.7393
	RF	0.7464	0.5033	0.7366	0.7685	0.7525
	FKeERF	0.7808	0.5644	0.7762	0.7866	0.7814
*S. cerevisiae*	KNN	0.632	0.277	0.6153	0.6622	0.6388
	SVM	0.6337	0.2762	0.6292	0.6464	0.6378
	XGBoost	0.7819	0.5625	0.7584	0.8019	0.7801
	RF	0.8503	0.6972	0.8279	0.8713	0.8496
	FKeERF	0.8773	0.7589	0.8608	0.8998	0.8803
*M. musculus*	KNN	0.6854	0.3745	0.7052	0.6703	0.6877
	SVM	0.6844	0.3696	0.7072	0.6661	0.6866
	XGBoost	0.7564	0.5174	0.7698	0.7483	0.759
	RF	0.7574	0.5202	0.781	0.7388	0.7599
	FKeERF	0.7787	0.5571	0.7785	0.7782	0.7784

**Table 7:** Performance of different classifiers on two species independent testing datasets

Species	Classifier	Independent testing				
		ACC	MCC	SN	SP	AUC
*H. sapiens*	KNN	0.645	0.3353	0.6602	0.6715	0.6659
	SVM	0.6499	0.3114	0.6519	0.6543	0.6531
	XGBoost	0.86	0.7198	0.8526	0.8614	0.857
	RF	0.9	0.7959	0.8732	0.925	0.8991
	FKeERF	0.9199	0.8504	0.9011	0.9497	0.9254
*S. cerevisiae*	KNN	0.7349	0.5039	0.7029	0.8061	0.7545
	SVM	0.76	0.5073	0.7258	0.7764	0.7511
	XGBoost	0.885	0.7588	0.9075	0.8592	0.8834
	RF	0.9349	0.8685	0.9141	0.9546	0.9343
	FKeERF	0.945	0.8898	0.9329	0.9608	0.9468

As can be seen from Table 6, FKeERF achieved the overall best performance on three species in terms of ACC, MCC, and AUC, compared to the other four traditional classifiers on the same training data set. For *H. sapiens*, FKeERF performed best in ACC, MCC and AUC, with its ACC, MCC and AUC 3.44, 6.11 and 2.89% higher than the next best RF, respectively. XGBoost performed worse than RF, but better than SVM, and KNN performed the worst. On the training set of *S. cerevisiae*, FKeERF obtained the best performance on ACC, MCC and AUC, 87.73, 75.89 and 88.03%, respectively, which was higher than the best performance value of the second best RF. XGBoost performed third best, with SVM and KNN performing relatively close but not as well as XGBoost. FKeERF continues to perform best on the training set of *M. musculus*, with ACC, MCC and AUC 2.13, 3.69 and 1.85 higher than RF, respectively. XGBoost and RF have similar performance, both better than KNN and SVM. FKeERF also achieved very competitive performance on independent testing datasets, outperforming the second best RF on both *H. sapiens*- and *S. cerevisiae*-independent testing set. XGBoost performed better than SVM and KNN, with KNN performing the worst.

By comparing the test results, it can be seen that our method FKeERF is superior to existing traditional classifiers in most evaluation indexes. In addition, it can be seen that ensemble learning methods perform better in the task of identifying $\Psi $ sites on the whole.

### Comparison with other existing methods

In this section, we thoroughly assess and compare the predictive performance of PseU-FKeERF with other methods, using the same benchmark training and independent testing data sets utilized by several previous state-of-the-art methods. Tables 8 and 9 summarize the performance comparisons between the PseU-FKeERF and several state-of-the-art predictors including iRNA-PseU [22], PseUI [23], iPseU-CNN [24], XG-PseU [25], EnsemPseU [26], RF-PseU [27], MU-PseUDeep [28], Porpoise [29], PsoEL-PseU [31] and PseUdeep [30] on the same benchmark training and independent testing dataset, respectively. Compared to other existing methods using the same training dataset, PseU-FKeERF performed better on two important measures for three species, namely ACC and MCC. For *H. sapiens*, PseU-FKeERF has an ACC and MCC of 78.08 and 56.44%, respectively, second only to the first best Porpoise. For *S. cerevisiae*, PseU-FKeERF achieved the best performance on both ACC and MCC, 6.04 and 12.51% higher than the next best method, Porpoise, respectively. For *M. musculus*, PseU-FKeERF also had the best performance, with ACC and MCC 0.12 and 0.16% higher, respectively, than the next best method Porpoise. On the three training data sets, the AUC of PseU-FKeERF is higher than that of other advanced methods, indicating that PseU-FKeERF has stronger classification ability between positive and negative classes.

**Table 8:** Performance comparison of PseU-FKeERF and 10 state-of-the-art methods on the same benchmark training datasets

Species	Methods	10-fold cross-validation				
		ACC (%)	MCC (%)	SN (%)	SP (%)	AUC (%)
*H. sapiens*	iRNA-PseU	60.40	21.00	61.01	59.80	64.00
	PseUI	64.24	28.00	64.85	63.64	68.00
	iPseU-CNN	66.68	34.00	65.00	68.78	/
	XG-PseU	65.44	31.00	63.64	67.24	70.00
	EnsemPseU	66.28	33.00	63.46	69.09	70.00
	RF-PseU	64.30	29.00	66.10	62.60	70.00
	MU-PseUDeep	72.60	52.40	70.90	81.00	/
	Porpoise	78.53	58.45	89.11	67.94	/
	PsoEL-PseU	70.80	42.00	66.90	74.70	/
	PseUdeep	66.99	35.00	74.47	60.71	74.00
	PseU-FKeERF	78.08	56.44	77.62	78.66	78.14
*S. cerevisiae*	iRNA-PseU	64.49	29.00	64.65	64.33	81.00
	PseUI	65.13	30.00	62.74	67.52	69.00
	iPseU-CNN	68.15	37.00	66.36	70.45	/
	XG-PseU	68.15	37.00	66.84	69.45	74.00
	EnsemPseU	74.16	49.00	73.88	74.45	78.60
	RF-PseU	74.80	49.00	77.20	72.40	81.00
	MU-PseUDeep	76.80	54.60	74.20	79.80	/
	Porpoise	81.69	63.38	81.21	82.17	/
	PsoEL-PseU	80.30	62.00	69.10	91.40	/
	PseUdeep	72.73	45.00	61.75	78.13	74.00
	PseU-FKeERF	87.73	75.89	86.08	89.98	88.03
*M. musculus*	iRNA-PseU	69.07	38.00	73.31	64.83	75.00
	PseUI	70.44	41.00	74.58	66.31	77.00
	iPseU-CNN	71.81	44.00	74.79	69.11	/
	XG-PseU	72.03	45.00	76.48	67.57	77.00
	EnsemPseU	73.85	48.00	75.43	72.25	77.50
	RF-PseU	74.80	50.00	73.10	76.50	79.60
	MU-PseUDeep	76.00	53.70	80.00	73.00	/
	Porpoise	77.75	55.55	77.83	77.67	/
	PsoEL-PseU	76.50	53.00	82.20	70.80	/
	PseUdeep	72.45	44.00	66.70	77.36	77.00
	PseU-FKeERF	77.87	55.71	77.85	77.82	77.84

**Table 9:** Comparison of the performance of PseU-FKeERF and other state-of-the-art methods on the same independent testing datasets

Species	Classifier	Independent testing				
		ACC (%)	MCC (%)	SN (%)	SP (%)	AUC (%)
*H. sapiens*	iRNA-PseU	61.50	23.00	58.00	65.00	/
	PseUI	65.00	31.00	64.85	68.00	/
	iPseU-CNN	69.00	40.00	77.72	60.81	/
	XG-PseU	67.50	35.00	68.00	67.00	/
	EnsemPseU	69.50	39.00	73.00	66.00	/
	RF-PseU	75.00	50.00	78.00	72.00	80.00
	Porpoise	77.35	55.13	82.30	72.40	/
	PsoEL-PseU	75.50	51.00	76.00	75.00	/
	PseUdeep	66.18	33.00	73.53	58.82	72.00
	PseU-FKeERF	91.99	85.04	90.11	94.97	92.54
*S. cerevisiae*	iRNA-PseU	60.00	20.00	63.00	57.00	/
	PseUI	68.50	37.00	65.00	72.00	/
	iPseU-CNN	73.50	47.00	68.76	77.82	/
	XG-PseU	71.00	42.14	75.00	67.00	/
	EnsemPseU	75.00	51.00	85.00	65.00	/
	RF-PseU	77.00	54.00	75.00	79.00	83.80
	Porpoise	83.50	67.27	88.00	79.00	/
	PsoEL-PseU	82.00	64.00	83.00	81.00	/
	PseUdeep	80.88	62.00	77.45	84.31	90.90
	PseU-FKeERF	94.50	88.98	93.29	96.08	94.68

The excellent performance of PseU-FKeERF on the benchmark training dataset shows that the fuzzy subset and kernel method used to predict the class module can improve the effectiveness of model training. We further conducted independent tests to evaluate and validate the trained model using independent testing data sets. Using the same independent testing data set, we assessed and contrasted the predictive performance of PseU-FKeERF with that of other current methods. The results are summarized in Table 9. The PseU-FKeERF model also achieves good performance on two independent testing sets. For both *H. sapiens* and *S. cerevisiae*, PseU-FKeERF achieved the best ACC, with improvements of 14.64 and 11.0%, respectively. The AUC of PseU-FKeERF on two independent test datasets is significantly higher than that of other methods, which further verifies that PseU-FKeERF has better generalization ability. PseU-FKeERF also has high F1 score and AUPR values on the training set and independent testing set, as shown in the Supplementary Tables S3 and S4.

In conclusion, PseU-FKeERF has some advantages and achieves better prediction performance when compared to other current models. PseU-FKeERF is a competitive model for the identification of RNA $\Psi $ sites in *H. sapiens*, *S. cerevisiae* and *M. musculus*.

## CONCLUSION

In this study, we developed a new model, called PseU-FKeERF, to achieve more accurate and stable predictions of $\Psi $ sites in *H. sapiens*, *S. cerevisiae* and *M. musculus*. In order to select the best features, we comprehensively evaluated the feature coding schemes of various RNA sequences, and selected four coding schemes with relatively better performance, including Binary, PSTNPss, NCP and PseKNC, for feature combination. Then, fuzzy mean clustering and Gaussian fuzzy membership are used to construct multiple feature subsets to form a new fuzzy feature set, which is input into the KeERF method for category prediction. Performance comparisons through cross-validation and independent testing show that the PseU-FKeERF performs better than several state-of-the-art predictors.

Although our model has proven excellent performance, there is still room for further improvement. First, compared with the traditional RF model, the running time of our model is relatively long, as shown in Supplementary Tables S5 and S6. Secondly, compared with previous advanced models, the performance of our model on the *H. sapiens* training dataset needs to be further improved. Our model performs better on the other four datasets, but slightly worse than Porpoise on the $H\_990$ data set. We analyzed that the reason for this problem may lie in the particularity of the data set. This data set may have special properties or feature distributions that make the proposed method impossible to generalize effectively on this particular data set. In addition, in some cases, a particular data set may give rise to accidental results, causing the proposed method to perform poorly on that data set. This may be due to the randomness of the data set or the influence of a particular sample. Finally, our model has the potential to be further improved by exploring more emerging technologies [56–60], such as analyzing biological sequences at sequence level and residue level based on relevant platforms, identifying sites using improved neural network models, applying fuzzy inference systems and multi-label learning, etc., with the aim of improving its overall performance. Despite these limitations, our study provides a new method for accurately identifying $\Psi $ sites in RNA sequences, and provides certain references for clinical research and related drug development.

**Key Points**

- We use fuzzy mean clustering and Gaussian fuzzy membership to construct fuzzy subsets, form a new fuzzy feature set and expand the original feature space.
- We combine kernel method with evidence Random Forest ERF, improve the category prediction method and develop a new classifier KeERF.
- We combine fuzzy logic with ensemble learning model to improve the accuracy of the model to identify $\Psi $ sites, so as to provide reference for practical applications.

## Supplementary Material

Supplementary_Material_bbae169

## Data Availability

The data sets and source codes are available at https://github.com/xgcms/PseU-FKeERF.

## Author Biographies

**Mingshuai Chen** is currently working toward the master's student with Institute of Fundamental and Frontier Sciences, University of Electronic Science and Technology of China. His research interests include bioinformatics and machine learning.

**Mingai Sun** is the attending physician, graduated from Jiamusi University with a bachelor's degree and a bachelor's degree, has been engaged in clinical work for more than 27 years. She has studied at the Ninth People's Hospital affiliated with Shanghai Jiao Tong University and is skilled in teeth whitening, aesthetic restoration, diagnosis and treatment of pulp disease, periapical disease, periodontal disease, and common oral diseases; Especially for complete dentures, pediatric dental diseases, and dental implants. She has won two Class III promotion projects for new medical technologies in Heilongjiang Province, one second prize and one third prize of the Harbin Medical and Health New Technology Application Award. As the corresponding author, she has published multiple SCI papers.

**Xi Su** is the president of the Foshan Women and Children Hospital, chief physician, and a master postgraduate advisor of Sun Yat-sen University (Guangzhou, China) and Southern Medical University (Guangzhou, China).

**Prayag Tiwari (Member, IEEE)** received his Ph.D degree from the University of Padova, Italy. He is currently working as a Postdoctoral Researcher at the Halmstad University. He has several publications in top journals and conferences including, Neural Networks, Information Fusion, IPM, IJCV, IEEE TNNLS, IEEE TFS, IEEE TII, ACM TOIT, CIKM, SIGIR, AAAI, etc. His research interests include Machine Learning, Deep Learning, Quantum Machine Learning, Information Retrieval, Healthcare, and IoT.

**Yijie Ding (Member, CAAI and IEEE)** received the PhD degree from the School of Computer Science and Technology, Tianjin University in 2018. Currently, he is an associate professor in University of Electronic Science and Technology of China. His research interests include bioinformatics and machine learning.

## References

[1] Ge JH , Yu YT. RNA pseudouridylation: new insights into an old modification. Trends Biochem Sci 2013;38(4):210–8. 23391857 10.1016/j.tibs.2013.01.002 PMC3608706

[2] Charette M , Gray MW. Pseudouridine in RNA: what, where, how, and why. IUBMB Life 2000;49(5):341–51. 10902565 10.1080/152165400410182

[3] Zou Q , Xing P, Wei L, Liu B. Gene2Vec: gene subsequence embedding for prediction of mammalian N-6-methyladenosine sites from mRNA. RNA 2019;25(2):205–18. 30425123 10.1261/rna.069112.118 PMC6348985

[4] Boo SH , Kim YK. The emerging role of RNA modifications in the regulation of mRNA stability. Exp Mol Med 2020;52(3):400–8. 32210357 10.1038/s12276-020-0407-z PMC7156397

[5] Basak A , Query CC. A Pseudouridine residue in the spliceosome core is part of the filamentous growth program in yeast. Cell Rep 2014;8(4):966–73. 25127136 10.1016/j.celrep.2014.07.004 PMC4425566

[6] Jack K , Bellodi C, Landry DM, et al. rRNA Pseudouridylation defects affect ribosomal ligand binding and translational fidelity from yeast to human cells. Mol Cell 2011;44(4):660–6. 22099312 10.1016/j.molcel.2011.09.017 PMC3222873

[7] Jin J , Yu Y, Wang R, et al. iDNA-ABF: multi-scale deep biological language learning model for the interpretable prediction of DNA methylations. Genome Biol 2022;23(1):1–23. 36253864 10.1186/s13059-022-02780-1 PMC9575223

[8] Carlile TM , Rojas-Duran MF, Zinshteyn B, et al. Pseudouridine profiling reveals regulated mRNA pseudouridylation in yeast and human cells. Nature 2014;515(7525):143–6. 25192136 10.1038/nature13802 PMC4224642

[9] Schwartz S , Bernstein DA, Mumbach MR, et al. Transcriptome-wide mapping reveals widespread dynamic-regulated pseudouridylation of ncRNA and mRNA. Cell 2014;159(1):148–62. 25219674 10.1016/j.cell.2014.08.028 PMC4180118

[10] Mei YP , Liao JP, Shen J, et al. Small nucleolar RNA 42 acts as an oncogene in lung tumorigenesis. Oncogene 2012;31(22):2794–804. 21986946 10.1038/onc.2011.449 PMC4966663

[11] Cao C , Wang J, Kwok D, et al. webTWAS: a resource for disease candidate susceptibility genes identified by transcriptome-wide association study. Nucleic Acids Res 2022;50(D1):D1123–30. 34669946 10.1093/nar/gkab957 PMC8728162

[12] Tang W , Wan S, Yang Z, et al. Tumor origin detection with tissue-specific miRNA and DNA methylation markers. Bioinformatics 2018;34(3):398–406. 29028927 10.1093/bioinformatics/btx622

[13] Chen L , Yu L, Gao L. Potent antibiotic design via guided search from antibacterial activity evaluations. Bioinformatics 2023;39(2):btad059. 10.1093/bioinformatics/btad059 PMC9897189 36707990

[14] Alzheimer's Association. 2023 Alzheimer’s disease facts and figures. Alzheimers Dement 2023;19(4):1598–695. 36918389 10.1002/alz.13016

[15] Hu Y , Sun JY, Zhang Y, et al. rs1990622 variant associates with Alzheimer’s disease and regulates TMEM106B expression in human brain tissues. BMC Med 2021;19(1):11. 33461566 10.1186/s12916-020-01883-5 PMC7814705

[16] Hu Y , Zhang H, Liu B, et al. rs34331204 regulates TSPAN13 expression and contributes to Alzheimer’s disease with sex differences. Brain 2020;143(11):e95–e95. 10.1093/brain/awaa302 PMC7719023 33175954

[17] Hu Y , Zhang Y, Zhang H, et al. Mendelian randomization highlights causal association between genetically increased C-reactive protein levels and reduced Alzheimer’s disease risk. Alzheimers Dement 2022;18(10):2003–6. 35598332 10.1002/alz.12687

[18] Hu Y , Zhang Y, Zhang H, et al. Cognitive performance protects against Alzheimer’s disease independently of educational attainment and intelligence. Mol Psychiatry 2022;27(10):4297–306. 35840796 10.1038/s41380-022-01695-4

[19] Li YH , Zhang GG, Cui QH. PPUS: a web server to predict PUS-specific pseudouridine sites. Bioinformatics 2015;31(20):3362–4. 26076723 10.1093/bioinformatics/btv366

[20] Zhang HY , Zou Q, Ju Y, et al. Distance-based support vector machine to predict DNA N6-methyladenine modification. Current Bioinformatics 2022;17(5):473–82.

[21] Wang Y , Zhai Y, Ding Y, Zou Q. SBSM-pro: support bio-sequence machine for proteins. arXiv preprint arXiv:230810275. 2023.

[22] Chen W , Tang H, Ye J, et al. iRNA-PseU: identifying RNA pseudouridine sites. Mol Ther-Nucleic Acids 2016;5:9. 10.1038/mtna.2016.37 PMC5330936 28427142

[23] He JJ , Fang T, Zhang ZZ, et al. PseUI: pseudouridine sites identification based on RNA sequence information. BMC Bioinform 2018;19:11. 10.1186/s12859-018-2321-0 PMC6114832 30157750

[24] Tahir M , Tayara H, Chong KT. iPseU-CNN: identifying RNA pseudouridine sites using convolutional neural networks. Mol Ther-Nucleic Acids 2019;16:463–70. 31048185 10.1016/j.omtn.2019.03.010 PMC6488737

[25] Liu KW , Chen W, Lin H. XG-PseU: an eXtreme gradient boosting based method for identifying pseudouridine sites. Mol Genet Genomics 2020;295(1):13–21. 31392406 10.1007/s00438-019-01600-9

[26] Bi Y , Jin D, Jia CZ. EnsemPseU: identifying pseudouridine sites with an ensemble approach. IEEE Access 2020;8:79376–82.

[27] Lv ZB , Zhang J, Ding H, Zou Q. RF-PseU: a random Forest predictor for RNA Pseudouridine sites. Front Bioeng Biotechnol 2020;8:10. 32175316 10.3389/fbioe.2020.00134 PMC7054385

[28] Khan SM , He F, Wang DL, et al. MU-PseUDeep: a deep learning method for prediction of pseudouridine sites. Comput Struct Biotechnol J 2020;18:1877–83. 32774783 10.1016/j.csbj.2020.07.010 PMC7387732

[29] Li FY , Guo XD, Jin PP, et al. Porpoise: a new approach for accurate prediction of RNA pseudouridine sites. Brief Bioinform 2021;22(6):12. 10.1093/bib/bbab245 PMC8575008 34226915

[30] Zhuang JJ , Liu DY, Lin M, et al. PseUdeep: RNA pseudouridine site identification with deep learning algorithm. Front Genet 2021;12:9. 10.3389/fgene.2021.773882 PMC8637112 34868261

[31] Wang X , Lin X, Wang R, et al. A feature fusion predictor for RNA pseudouridine sites with particle swarm optimizer based feature selection and ensemble learning approach. Curr Issues Mol Biol 2021;43(3):1844–58. 34889887 10.3390/cimb43030129 PMC8929013

[32] Deng ZH , Jiang YZ, Choi KS, et al. Knowledge-leverage-based TSK fuzzy system modeling. IEEE Trans Neural Netw Learn Syst 2013;24(8):1200–12. 24808561 10.1109/TNNLS.2013.2253617

[33] Dempster AP . Upper and lower probabilities induced by a multivalued mapping. *Ann Math Stat* 1967;38(2):325–39.

[34] Shafer G . A Mathematical Theory of Evidence, Vol. 42. Princeton University Press, London, 1976.

[35] Jousselme AL , Grenier D, Bosse E. A new distance between two bodies of evidence. Inf Fusion 2001;2(2):91–101.

[36] Martin A (ed). Conflict Management in Information Fusion with Belief Functions. Cham: Springer International Publishing, 2019, 79–97.

[37] Chen Z , Zhao P, Li FY, et al. iLearn: an integrated platform and meta-learner for feature engineering, machine-learning analysis and modeling of DNA, RNA and protein sequence data. Brief Bioinform 2020;21(3):1047–57. 31067315 10.1093/bib/bbz041

[38] Chen Z , Zhao P, Li C, et al. iLearnPlus: a comprehensive and automated machine-learning platform for nucleic acid and protein sequence analysis, prediction and visualization. Nucleic Acids Res 2021;49(10):e60. 33660783 10.1093/nar/gkab122 PMC8191785

[39] Li H , Pang Y, Liu B. BioSeq-BLM: a platform for analyzing DNA, RNA, and protein sequences based on biological language models. Nucleic Acids Res 2021;49(22):e129. 34581805 10.1093/nar/gkab829 PMC8682797

[40] Ao C , Ye X, Sakurai T, et al. m5U-SVM: identification of RNA 5-methyluridine modification sites based on multi-view features of physicochemical features and distributed representation. BMC Biol 2023;21(1):14. 10.1186/s12915-023-01596-0 PMC10127088 37095510

[41] Su W , Xie XQ, Liu XW, et al. iRNA-ac4C: a novel computational method for effectively detecting N4-acetylcytidine sites in human mRNA. Int J Biol Macromol 2023;227:1174–81. 36470433 10.1016/j.ijbiomac.2022.11.299

[42] Wang R , Jiang Y, Jin J, et al. DeepBIO: an automated and interpretable deep-learning platform for high-throughput biological sequence prediction, functional annotation and visualization analysis. Nucleic Acids Res 2023;51(7):3017–29. 36796796 10.1093/nar/gkad055 PMC10123094

[43] Wang L , Ding Y, Tiwari P, et al. A deep multiple kernel learning-based higher-order fuzzy inference system for identifying DNA N4-methylcytosine sites. Inform Sci 2023;630:40–52.

[44] Hoarau A , Martin A, Dubois JC, Le Gall Y. Evidential random forests. Exp Syst Appl 2023;230:120652.

[45] Scornet E . Random forests and kernel methods. IEEE Trans Inf Theory 2016;62(3):1485–500.

[46] Li H , Liu B. BioSeq-diabolo: biological sequence similarity analysis using diabolo. PLoS Comput Biol 2023;19(6):e1011214. 37339155 10.1371/journal.pcbi.1011214 PMC10313010

[47] Sun WJ , Li JH, Liu S, et al. RMBase: a resource for decoding the landscape of RNA modifications from high-throughput sequencing data. Nucleic Acids Res 2016;44(D1):D259–65. 26464443 10.1093/nar/gkv1036 PMC4702777

[48] Zou Y , Ding YJ, Tang JJ, et al. FKRR-MVSF: a fuzzy kernel ridge regression model for identifying DNA-binding proteins by multi-view sequence features via Chou’s five-step rule. Int J Mol Sci 2019;20(17):14. 10.3390/ijms20174175 PMC6747228 31454964

[49] Ding Y , Tang J, Guo F. Identification of drug-target interactions via multi-view graph regularized link propagation model. Neurocomputing 2021;461:618–31.

[50] Guo F , Ding Y, Li Z, Tang J. Identification of protein-protein interactions by detecting correlated mutation at the interface. J Chem Inf Model 2015;55(9):2042–9. 26284382 10.1021/acs.jcim.5b00320

[51] Ding Y , He W, Tang J, et al. Laplacian regularized sparse representation based classifier for identifying DNA N4-methylcytosine sites via L2, 1/2-matrix norm. IEEE/ACM Trans Comput Biol Bioinform 2023;20(1):500–11. 10.1109/TCBB.2021.3133309 34882559

[52] Yan K , Lv H, Guo Y, et al. sAMPpred-GAT: prediction of antimicrobial peptide by graph attention network and predicted peptide structure. Bioinformatics 2023;39(1):btac715. 36342186 10.1093/bioinformatics/btac715 PMC9805557

[53] Tang Y , Pang Y, Liu B. IDP-Seq2Seq: identification of intrinsically disordered regions based on sequence to sequence learning. Bioinformatics 2021;36(21):5177–86. 32702119 10.1093/bioinformatics/btaa667

[54] Zou X , Ren L, Cai P, et al. Accurately identifying hemagglutinin using sequence information and machine learning methods. Front Med (Lausanne) 2023;10:1281880. 38020152 10.3389/fmed.2023.1281880 PMC10644030

[55] Zhu W , Yuan SS, Li J, et al. A first computational frame for recognizing heparin-binding. Protein Diagn (Basel) 2023;13(14):14. 10.3390/diagnostics13142465 PMC10377868 37510209

[56] Ding Y , Tiwari P, Guo F, Zou Q. Shared subspace-based radial basis function neural network for identifying ncRNAs subcellular localization. Neural Netw 2022;156:170–8. 36274524 10.1016/j.neunet.2022.09.026

[57] Liu B , Gao X, Zhang H. BioSeq-Analysis2.0: an updated platform for analyzing DNA, RNA and protein sequences at sequence level and residue level based on machine learning approaches. Nucleic Acids Res 2019;47(20):e127. 31504851 10.1093/nar/gkz740 PMC6847461

[58] Ding Y , Tiwari P, Guo F, Zou Q. Multi-correntropy fusion based fuzzy system for predicting DNA N4-methylcytosine sites. Inf Fusion 2023;100:101911.

[59] Wang H , Ding Y, Tang J, et al. Identify RNA-associated subcellular localizations based on multi-label learning using Chou’s 5-steps rule. BMC Genomics 2021;22(1):1–14. 33451286 10.1186/s12864-020-07347-7 PMC7811227

[60] Ding Y , Tiwari P, Zou Q, et al. C-loss based higher order fuzzy inference systems for identifying DNA N4-methylcytosine sites. IEEE Trans Fuzzy Syst 2022;30(11):4754–65.

