# Supplementary material for: Fuzzy kernel evidence Random Forest for identifying pseudouridine sites
Source: Brief Bioinform. 2024 Apr 15;25(3):bbae169. doi: 10.1093/bib/bbae169 (PMC11018548; doi:10.1093/bib/bbae169)
Supplement: Supplementary_Material_bbae169 [file supplementary_material_bbae169.docx]

**Supplementary Material**

**Contents**

Tables:

[**Table S1**. Advantages and limitations of existing advanced methods for identifying Ψ sites 2](#_Toc161149313)

[**Table S2.** Optimization parameters of other advanced models 4](#_Toc161149314)

[**Table S3**. The F1 score and AUPR of PseU-FKeERF on three training datasets 7](#_Toc161149315)

[**Table S4**. The F1 score and AUPR of PseU-FKeERF on two independent testing datasets 7](#_Toc161149316)

[**Table S5**. Running time comparison of FKeERF and RF on three training datasets 8](#_Toc161149317)

[**Table S6**. Running time comparison of FKeERF and RF on two independent testing datasets 8](#_Toc161149318)

# **Table S1**. Advantages and limitations of existing advanced methods for identifying Ψ sites

| **Tool** | **Advantage** | **Limitation** |
| --- | --- | --- |
| PPUS | An effective method for predicting Ψ sites was developed for the first time. | Few features, only binary features are used.  Only Ψ sites in H.sapiens and S.cerevisiae can be identified |
| iRNA-PseU | Ψ sites in H. sapiens, S. cerevisiae and M. musculus can be identified. | Relatively few feature types are used, and the accuracy of H.sapiens training set needs to be improved |
| PseUI | Five different types of features are combined while a sequential forward feature selection strategy is used. | The accuracy of H. sapiens and S. cerevisiae species training sets is relatively low. |
| iPseU-CNN | Deep learning techniques are used. | No open source code is provided |
| XG-PseU | The optimal features obtained by forward feature selection and incremental feature selection are used, and XGBoost is used to identify Ψ sites. | The accuracy of H. sapiens and S. cerevisiae species training sets was not significantly improved. |
| EnsemPseU | Chi-square feature selection is used to remove redundant information, and ensemble learning algorithms are used to construct predictive models. | KNN performs poorly in single classifiers. The performance of not integrating KNN into the model is not considered. |
| RF-PseU | The optical gradient elevator algorithm and incremental feature selection strategy are used to select the optimal feature space. | Poor performance on the H.sapiens species training set. |
| MU-PseUDeep | The secondary structure context of an  mRNA fragment is used as an input feature | Relatively few feature types are used |
| Porpoise | The latest feature coding schemes and machine learning algorithms are systematically analyzed and evaluated. | The accuracy of M. musculus species training set was not significantly improved. |
| PseUdeep | Build models using deep learning techniques. | Fewer feature coding schemes are used. |
| PsoEL-PseU | A thorough search selection algorithm is used to eliminate redundant features and a parallel fusion strategy is adopted. | The calculation cost is high and the resource consumption is large. |

# **Table S2.** Optimization parameters of other advanced models

| **Methods** | **Algorithm** | **Optimized parameters** |
| --- | --- | --- |
| iRNA-PseU | SVM | The regularization parameter $C$ and the kernel width parameter $\gamma$  $\left\{ \begin{aligned} 2^{-5}\leq C\leq2^{15} with step of 2 \\ 2^{-15}\leq\gamma\leq2^{-5} with step of 2^{-1} \end{aligned} \right.$ |
| PseUI | SVM | The SVM parameter $g$ from $2^{-15}$ to $2^{-5}$ and parameter $c$ from $2^{-5}$ to $2^{15}$ with a step of 2. Finally, the parameters $g$ and $c$ were set at 0.01562 and 2 for H. sapiens, 0.0003 and 32,768 for S. cerevisiae, and 0.00098 and 4 for M.musculus, respectively. |
| iPseU-CNN | CNN | The ranges of the tuned hyper-parameters   \| **Hyper-Parameter** \| **Range** \| \| --- \| --- \| \| Convolution layers \| [1,2] \| \| Filters \| [5,7,9] \| \| Filter size \| [3,5,7] \| \| Stride \| [1,2] \| \| Dropout \| [0.25, 0.50] \| |
| XG-PseU | SVM | The parameters of the XGboost for the model of each species   \| **Species** \| **Estimators** \| **Depth** \| **Min_ child_ weight** \| **Subsample** \| **Colsample** \| \| --- \| --- \| --- \| --- \| --- \| --- \| \| H.sapiens \| 40 \| 3 \| 3 \| 0.7 \| 0.7 \| \| M.musculus \| 30 \| 3 \| 5 \| 0.6 \| 0.6 \| \| S.cerevisiae \| 30 \| 5 \| 3 \| 0.7 \| 0.7 \| |
| EnsemPseU | Ensemble | The number k of optimal features is 400. The parameter $\gamma$ of SVM$\in\{0.01, 0.02, 0.04, 0.06, 0.08, 0.1\}$, boosting the learning rate $r$ of XGBoost$\in\{0.1, 0.2, 0.3, 0.4, 0.5\},$ $k$neighbors of KNN$\in\{1, 3, 5, 7, 9\}$ and  the number of decision trees $n$ of RF$\in\{100, 200, 300, 400, 500\}$. |
| RF-PseU | RF | / |
| MU-PseUDeep | CNN | A stochastic gradient was used as the optimization algorithm with a learning rate of 0.0137. A binary cross-entropy was used as a loss function with an early-stop patience of 20. The batch size was 32 and the number of epochs was set to 500. The total number of trainable parameters in the network was 661,118. |
| Porpoise | Ensemble | Hyper-parameter values of the optimal stacked models   \| H_990 \| XGBoost \| Feature type \| PSTNPss \| \| --- \| --- \| --- \| --- \| \| Num of selected features \| 19 \| \| learning_rate \| 0.26288 \| \| n_estimators \| 1688 \| \| max_depth \| 7 \| \| subsample \| 0.650119 \| \| colsample_bytree \| 0.527786 \| \| gamma \| 0.045847 \| \| GBDT \| Feature type \| PSTNPss \| \| Num of selected features \| 19 \| \| learning_rate \| 0.236234 \| \| max_depth \| 4 \| \| subsample \| 0.62329 \| \| n_estimators \| 724 \| \| SVM \| Feature type \| Binary \| \| Num of selected features \| 7 \| \| C \| 26757.57219 \| \| kernel \| rbf \| \| gamma \| 0.002004565 \| \| probability \| True \| \| GaussianNB \| Feature type \| PseKNC \| \| Num of selected features \| 64 \| \| S_628 \| XGBoost \| Feature type \| PSTNPss \| \| Num of selected features \| 22 \| \| max_depth \| 3 \| \| n_estimators \| 2000 \| \| learning_rate \| 0.3 \| \| gamma \| 0.10089 \| \| subsample \| 1 \| \| colsample_bytree \| 1 \| \| GBDT \| Feature type \| PSTNPss \| \| Num of selected features \| 22 \| \| learning_rate, \| 0.3 \| \| max_depth \| 3 \| \| n_estimators \| 2000 \| \| subsample \| 1 \| \| M_944 \| XGBoost \| Feature type \| PSTNPss \| \| Num of selected features \| 15 \| \| learning_rate \| 0.3 \| \| max_depth \| 10 \| \| n_estimators \| 2000 \| \| gamma \| 0.022889 \| \| subsample \| 0.5 \| \| colsample_bytree \| 0.5 \| \| AdaBoost \| Feature type \| NCP \| \| Num of selected features \| 35 \| \| learning_rate \| 0.3 \| \| max_depth \| 3 \| \| n_estimators \| 500 \| |
| PsoEL-PseU | SVM | The regularization parameter $C$ and the kernel width parameter $\gamma$  $\left\{ \begin{aligned} 2^{-16}\leq C\leq2^{15} common ration of 2 \\ 2^{-16}\leq\gamma\leq2^{15} common ration of 2 \end{aligned} \right.$ |
| PseUdeep | Deep Learning | A binary cross-entropy is used as a loss function with an early  stop patience of 20. The batch size is 32, and the number of epochs is set to 200. For the stochastic gradient descent method, the Adam  optimization algorithm is selected here. The total number of  trainable parameters in the network is 165,365. |

# **Table S3**. The F1 score and AUPR of PseU-FKeERF on three training datasets

| Species | Cross-Validation | |
| --- | --- | --- |
|  | F1 score(%) | AUPR(%) |
| H.sapiens (H_990) | 79.61 | 77.81 |
| S.cerevisiae  (S_628) | 87.10 | 85.70 |
| M.musculus  (M_944) | 79.61 | 77.21 |

# **Table S4**. The F1 score and AUPR of PseU-FKeERF on two independent testing datasets

| Species | Independent Testing | |
| --- | --- | --- |
|  | F1 score(%) | AUPR(%) |
| H.sapiens (H_200) | 91.75 | 90.93 |
| S.cerevisiae  (S_200) | 94.02 | 92.43 |

# **Table S5**. Running time comparison of FKeERF and RF on three training datasets

| Species | Classifier | Running time (seconds) |
| --- | --- | --- |
| H. sapiens | RF | 675 |
|  | FKeERF | 909 |
| S. cerevisiae | RF | 441 |
|  | FKeERF | 741 |
| M. musculus | RF | 421 |
|  | FKeERF | 799 |

# **Table S6**. Running time comparison of FKeERF and RF on two independent testing datasets

| Species | Classifier | Running time (seconds) |
| --- | --- | --- |
| H. sapiens | RF | 24 |
|  | FKeERF | 17 |
| S. cerevisiae | RF | 25 |
|  | FKeERF | 18 |
